# Early depression screening and short-term functional outcome in hospitalized patients for acute ischemic stroke

**DOI:** 10.3389/fneur.2022.950045

**Published:** 2022-08-05

**Authors:** Minyoul Baik, Hyungwoo Lee, Il Hyung Lee, JoonNyung Heo, Hyo Suk Nam, Hye Sun Lee, Young Dae Kim

**Affiliations:** ^1^Department of Neurology, Yongin Severance Hospital, Yonsei University College of Medicine, Yongin, South Korea; ^2^Department of Neurology, Yonsei University College of Medicine, Seoul, South Korea; ^3^Integrative Research Center for Cerebrovascular and Cardiovascular Diseases, Seoul, South Korea; ^4^Biostatistics Collaboration Unit, Department of Research Affairs, Yonsei University College of Medicine, Seoul, South Korea

**Keywords:** ischemic stroke, transient ischemic attack, post-stroke depression, Patient Health Questionnaire-9, functional outcome

## Abstract

**Background:**

Patients with ischemic stroke are at high risk for post-stroke depression (PSD). There are limited data regarding the clinical impact of early PSD, assessed in hospitalized patients with acute ischemic stroke.

**Methods:**

This hospital-based observational cohort study included consecutive patients with acute ischemic stroke or transient ischemic attack between July 2019 and June 2021. In the study hospital, all admitted patients were systematically screened for depression. The depression was screened using the Patient Health Questionnaire-9 (PHQ-9), and PHQ-9 positivity indicated early PSD, which was defined as a score of >4. Logistic regression analyses were used to compare the rates of poor functional outcomes at 3 months in patients with and without PHQ-9 positivity.

**Results:**

Among 1339 patients admitted during the study period, 775 were included, with a median age of 68.0 years, and 316 (40.8%) were women. A total of 111 (14.3%) patients were PHQ-9 positive. History of cancer and early neurological deterioration were independently associated with PHQ-9 positivity. Poor functional outcomes at 3 months were observed in 147 patients (18.8%). PHQ-9 positivity independently showed a 2.2-fold increased risk of poor functional outcome at 3 months (Odds ratio 2.23; 95% confidence interval 1.05–4.73, *P* = 0.037).

**Conclusions:**

Patients with history of cancer and early neurological deterioration were at risk for early PSD. Early PSD was independently associated with poor functional outcomes at 3 months. The identification of early depression could offer opportunities for further questioning and exploration of symptoms, as well as interventions.

## Introduction

Post-stroke depression (PSD) is one of the most common complications of stroke affecting approximately one-third of stroke survivors (1). PSD is independently associated with poor functional outcomes and higher mortality after stroke (2, 3). The current guideline recommends routine screening for PSD among stroke survivors by administration of a structured depression inventory (4). However, optimal timing, setting, methods, and follow-up for PSD screening are currently unclear (4).

The importance of early recognition and treatment of PSD has led to recommendations for depression screening in the early stages of patients hospitalized for acute ischemic stroke (5). Although numerous validated tools for the diagnosis of depression have been used to screen PSD in the subacute or chronic phase, data on the usefulness of these screening measures during the acute in-hospital phase of stroke are still limited (2, 6).

The Patient Health Questionnaire 9 (PHQ-9) is a 9-item self-administered depression screening and diagnostic tool for PSD, which is well validated in the chronic phase of ischemic stroke survivors (2, 7). A previous meta-analysis assessing PSD screening tools (24 studies, *N* = 2907) found that the 20-item Center of Epidemiological Studies-Depression Scale (CES-D), 21-item Hamilton Depression Rating Scale (HAMD) and PHQ-9 had high sensitivity for detecting PSD (8). Hence, a recent scientific statement from American Heart Association/American Stroke Association suggested that PHQ-9 may be more pragmatic, while CES-D and HAMD may not be feasible in a busy clinical practice despite high sensitivity (2, 4). Recent randomized trials including Effects of fluoxetine on functional outcomes after acute stroke trial and Assessment of Fluoxetine in Stroke Recovery trial, which evaluated the effect of fluoxetine on functional outcome after acute stroke, also defined secondary outcomes of depressive mood based on PHQ results (9, 10). Several small studies have further attempted to define early PSD using the PHQ-9 in patients with stroke within the acute period (11–13).

In the study hospital, all hospitalized patients with acute ischemic stroke were systematically screened using the PHQ-9 during admission to detect early PSD (5). In this study, we attempted to define early PSD using the PHQ-9 in hospitalized patients with acute ischemic stroke or transient ischemic attack (TIA). We sought to explore factors associated with early PSD, as well as the relationship between early PSD and short-term outcomes.

## Methods

### Ethical approval

This study was approved by the institutional review board of Severance Hospital, Yonsei University Health System (no. 4–2021–1602), which waived the need for informed consent due to its retrospective nature.

### Patients and evaluation

This was a retrospective single-center based observational cohort study of patients with acute ischemic stroke or TIA who were prospectively registered in the Yonsei Stroke cohort (14). The registry enrolled consecutive patients with acute ischemic stroke or TIA within 7 days of stroke onset of those admitted to the stroke center.

All patients were thoroughly evaluated and managed based on current stroke guidelines (4). We collected patients' data on vascular risk factors such as hypertension, diabetes, dyslipidemia, body mass index (kg/m^2^), abdominal circumference, current smoking status, atrial fibrillation, cancer, and prior history of ischemic heart disease, stroke, or medication use. All patients underwent brain computed tomography (CT) and/or magnetic resonance imaging, cerebral angiographic studies (digital subtraction angiography, CT angiography, and/or magnetic resonance angiography), and chest radiography. Cardiac evaluations included 12-lead electrocardiography (ECG), continuous ECG monitoring during staying in a stroke unit and/or 24-h Holter monitoring; transesophageal echocardiography; and/or transthoracic echocardiography. During admission, the Korean version of the Mini-Mental State Examination (K-MMSE) was also evaluated along with education level (nil, primary school, or secondary school and above) and handedness (15). Stroke severity was assessed using the National Institute of Health Stroke Scale (NIHSS). Early neurological deterioration (END) was defined as any increase (≥1) in NIHSS score within 7 days after admission compared with baseline NIHSS score (16). After discharge, all patients were regularly followed up at 3 months, 1 year, and annually thereafter. Follow-up information was obtained by neurologists or clinical research assistants at the outpatient clinic through face-to-face interviews or through telephone interview using an in-house questionnaire (5). Poor functional outcome was defined as a modified Rankin scale (mRS) score of >2 at 3 months after stroke.

This study included patients with acute ischemic stroke or TIA who underwent PHQ-9 between July 2019 and June 2021. Patients with unknown mRS scores at 3 months were excluded from the study. Patients with depression before the onset of index stroke and/or prior antidepressant use, chronic alcoholism, or dementia were excluded. Further, patients without MMSE results were excluded from the study.

### Early depression screening

All patients admitted to the Severance Stroke Center were systematically screened for depression by trained nurses using the validated Korean version of PHQ-9 (17). PHQ-9 was usually performed within 3 days after admission especially during weekday daytime hours. For patients with END, PHQ was usually performed after the occurrence of END. If a patient could not undergo PHQ-9, the reason was consecutively clarified in the medical record as follows: poor systemic conditions, difficulties in communication due to aphasia and/or impaired mentality, poor cooperation, or other causes.

The PHQ-9 is a self-administered questionnaire completed by the patient (17, 18). It consists of nine questions [1. Little interest or pleasure in doing things? 2. Feeling down, depressed, or hopeless? 3. Trouble falling or staying asleep, or sleeping too much? 4. Feeling tired or having little energy? 5. Poor appetite or overeating? 6. Feeling bad about yourself - or that you are a failure or have let yourself or your family down? 7. Trouble concentrating on things, such as reading the newspaper or watching television? 8. Moving or speaking so slowly that other people could have noticed? or the opposite—being so fidgety or restless that you have been moving around a lot more than usual? 9. Thoughts that you would be better off dead, or of hurting yourself in some way?]. Each question is scored from 0 to 3 points based on the frequency of the specified symptoms in the preceding 2 weeks, reflecting the index stroke concomitantly. Scores range from 0 (no depression) to 27 (severe depression). PHQ-9 positivity, indicating early PSD, was defined as a score of >4 (more than mild depression) (7).

The corresponding action, including outpatient/inpatient psychiatric referral and initiation of antidepressant therapy, was decided by the treating physician. Escitalopram was usually prescribed to treat PSD in the study hospital (19). Besides treatment for depression, bupropion was used for smoking cessation (20).

### Statistical analyses

Statistical analysis was performed using R version 4.0.4 (http://www.R-project.org). Univariable analysis was performed using the independent *t-*test, analysis of variance, or Kruskal–Wallis test for continuous variables, and the chi-square test for categorical variables, as appropriate. Multivariable logistic regression analysis was performed to identify which variable was independently associated with PHQ-9 positive and whether PHQ-9 was independently associated with poor functional outcome at 3 months. Analyses were adjusted for age, sex, and variables (*P* < 0.05) in univariable analyses. Subgroup analyses were performed to investigate whether the association between PHQ-9 positivity and poor functional outcome at 3 months differed according to the following factors: median age, sex, and significant variables associated with PHQ-9 positivity. Considering the decreased number of patients in each subgroup, multivariable models were adjusted for age and sex in the subgroup analyses. Statistical significance was set at *P* < 0.05.

Furthermore, to reduce potential confounding effects of baseline characteristics, propensity score matching (PSM) analysis was employed. The propensity score for the predicted probability of PHQ-9 positive in each patient was estimated with a logistic regression model, using age, sex, education, Medicaid, right handedness, K-MMSE, hypertension, diabetes, dyslipidemia, previous stroke, ischemic heart disease, current smoker, atrial fibrillation, cancer, body mass index, prestroke mRS > 2, right hemispheric stroke, NIHSS at admission, intravenous tissue plasminogen activator, intra-arterial thrombectomy, and END. We created a propensity score-matched cohort by matching each patient with PHQ-9 positive to a patient with PHQ-negative (a 1:2 match). A nearest-neighbor-matching algorithm was used to match the participants based on a difference of 0.1 multiplied by the standard deviation for linearly transformed propensity scores (logit-transformation). Covariate balance was evaluated by using standardized mean differences, with a standardized difference of <0.2 for a given covariate being considered to have achieved covariate balance. In the matched cohort, patient characteristics between both groups were compared using linear mixed model or conditional logistic regression. Multivariable conditional logistic regression analyses were performed to evaluate whether PHQ-9 was associated with poor functional outcome at 3 months after propensity score matching. Analyses were adjusted for length of hospital stay, NIHSS at discharge, mRS at discharge >2, home discharge, and antidepressant except bupropion.

## Results

**Figure 1:**
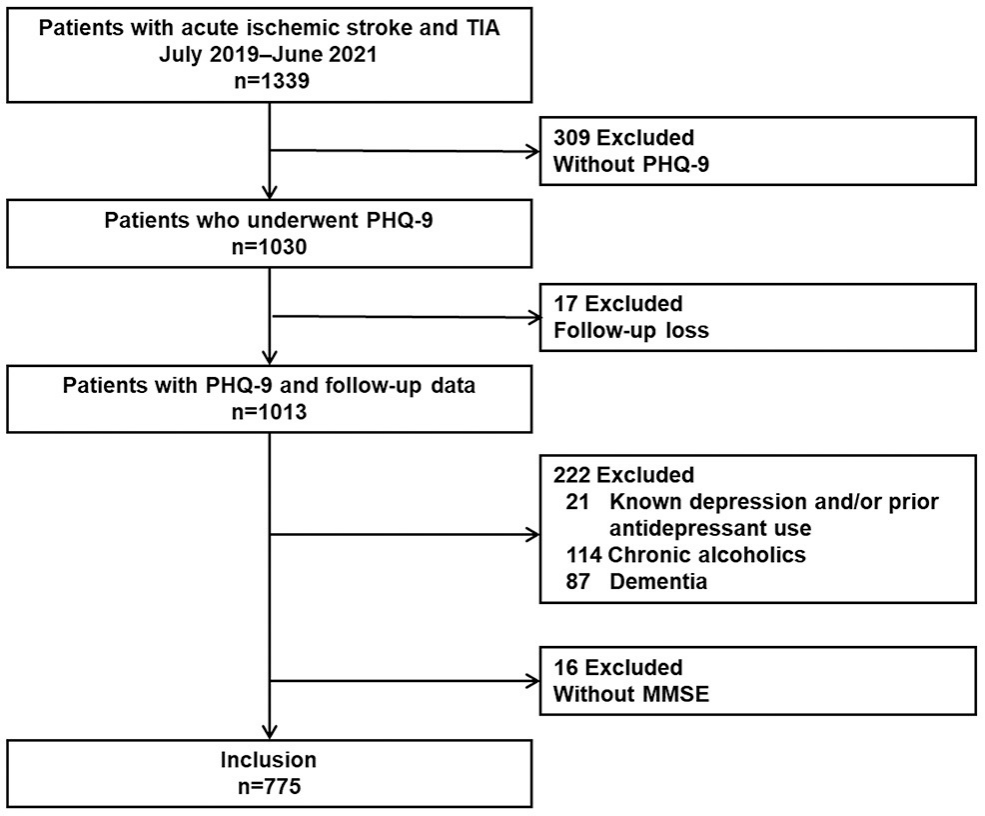
Patient selection. PHQ-9, Patient Health Questionnaire-9; MMSE, Mini-Mental State Exam.

During the study period, a total of 1339 consecutive patients with acute ischemic stroke or TIA were enrolled in the prospective registry, and 309 patients without PHQ-9 scores were excluded (poor systemic conditions [*N* = 33], communication difficulties [*N* = 205], poor cooperation [*N* = 31], or other causes [*N* = 40]). Seventeen patients had missing mRS scores at 3 months. Among 1,013 patients with PHQ-9 scores and proper follow-up data, we further excluded 21 patients with known depression and/or prior antidepressant use, 114 patients with chronic alcoholism, 87 patients with dementia, and 6 patients without K-MMSE results. Finally, 775 patients were included in this study (Figure 1). The median time interval between admission to PHQ-9 screening and stroke onset to PHQ-9 screening was 1 day (Inter-quartile range [IQR] 0–2.0 days) and 45 h (IQR 18–76 h), respectively.

Of the 775 patients enrolled (median age [IQR], 68.0 [59.0–77.0] years; and 316 [40.8%] women), 111 (14.3%) patients were PHQ-9 positive. The baseline characteristics according to the PHQ-9 are shown in Table 1. Both antidepressant and antidepressant except bupropion were more commonly prescribed in the PHQ-positive group. There was no difference in the bupropion description at discharge. Consultation to a psychiatrist during hospitalization was performed in 8 (1.0%) patients (2 [1.8%] in PHQ-9 positive vs. 6 [0.9%] in PHQ-9 negative group, *P* = 0.719).

**Table 1:** Comparison of characteristics of patients stratified based on PHQ-9 positivity.

	**Before Propensity score matching**			**After Propensity score matching**		
	**PHQ-9 Positive**	**PHQ-9 Negative**	***P* value**	**PHQ-9 Positive**	**PHQ-9 Negative**	***P* value**
	**(*N =* 111)**	**(*N =* 664)**		**(*N =* 100)**	**(*N =* 200)**	
***Demographic/Social variables***				
Age, years	65.0 [58.0–74.0]	69.0 [60.0–78.0]	0.041	64.5 [58.0–74.0]	67.0 [56.0–77.5]	0.761
Sex, female	51 (45.9)	265 (39.9)	0.274	45 (45.0)	94 (47.0)	0.745
Education			0.163			0.325
Nil	15 (13.5)	57 (8.6)		12 (12.0)	28 (14.0)	
Primary	21 (18.9)	108 (16.3)		17 (17.0)	46 (23.0)	
Secondary and above	75 (67.6)	499 (75.2)		71 (71.0)	126 (63.0)	
Medicaid	6 (5.4)	21 (3.2)	0.361	6 (6.0)	13 (6.5)	0.867
Right handed	104 (93.7)	630 (94.9)	0.782	97 (97.0)	186 (93.0)	0.125
MMSE	27.0 [24.0–29.0]	27.0 [25.0–29.0]	0.650	27.0 [22.5–29.0]	27.0 [24.0–29.0]	0.626
***Medical history***						
Hypertension	84 (75.7)	491 (73.9)	0.788	74 (74.0)	150 (75.0)	0.846
Diabetes	45 (40.5)	208 (31.3)	0.071	37 (37.0)	79 (39.5)	0.656
Dyslipidemia	44 (39.6)	258 (38.9)	0.959	38 (38.0)	75 (37.5)	0.932
Previous stroke	20 (18.0)	136 (20.5)	0.637	18 (18.0)	26 (13.0)	0.245
Ischemic heart disease	12 (10.8)	79 (11.9)	0.865	8 (8.0)	18 (9.0)	0.767
Current smoker	22 (19.8)	119 (17.9)	0.729	19 (19.0)	37 (18.5)	0.912
Atrial fibrillation	18 (16.2)	114 (17.2)	0.912	15 (15.0)	29 (14.5)	0.908
Cancer	28 (25.2)	92 (13.9)	0.003	24 (24.0)	39 (19.5)	0.362
Body mass index	24.0 [21.6–26.4]	24.0 [22.0–25.9]	0.820	23.9 [21.1–26.3]	24.1 [22.0–25.9]	0.455
Prestroke mRS	0.0 [0.0–0.0]	0.0 [0.0–0.0]	0.647	0.0 [0.0–0.0]	0.0 [0.0–0.0]	0.553
> 2	4 (3.6)	14 (2.1)	0.530	4 (4.0)	5 (2.5)	0.461
***Stroke variables***						
Right hemisphere	63 (56.8)	364 (54.8)	0.782	56 (56.0)	113 (56.5)	0.936
NIHSS at admission	3.0 [1.0–5.0]	2.0 [1.0–5.0]	0.175	3.0 [1.0–5.0]	3.0 [1.0–5.0]	0.715
IV tPA	8 (7.2)	59 (8.9)	0.689	7 (7.0)	12 (6.0)	0.741
IAT	3 (2.7)	46 (6.9)	0.138	3 (3.0)	6 (3.0)	>0.999
Length of hospital stay, days	5.0 [4.0–7.0]	4.0 [3.0–6.5]	0.034	5.0 [4.0–7.0]	5.0 [4.0–7.5]	0.962
END	34 (30.6)	140 (21.1)	0.035	27 (27.0)	58 (29.0)	0.707
NIHSS at discharge	2.0 [1.0–4.0]	1.0 [0.0–2.0]	<0.001	2.0 [0.0–3.5]	1.0 [0.0–3.0]	0.012
mRS at discharge	2.0 [1.0–3.0]	1.0 [1.0–2.0]	<0.001	2.0 [1.0–3.0]	1.0 [1.0–3.0]	0.018
>2	34 (30.6)	145 (21.8)	0.056	30 (30.0)	52 (26.0)	0.439
Discharge destination			0.003			0.017
Home	70 (63.1)	520 (78.3)		63 (63.0)	146 (73.0)	
Transfer to RH	15 (13.5)	65 (9.8)		13 (13.0)	30 (15.0)	
Transfer to other department	10 (9.0)	33 (5.0)		10 (10.0)	9 (4.5)	
Transfer to other hospital	16 (14.4)	46 (6.9)		14 (14.0)	15 (7.5)	
All antidepressant	34 (30.6)	131 (19.7)	0.013	29 (29.0)	44 (22.0)	0.181
Antidepressant other than bupropion	16 (14.4)	19 (2.9)	<0.001	13 (13.0)	10 (5.0)	0.013
SSRI	11 (68.8)	17 (89.5)		10 (76.9)	9 (90.0)	
SNRI	2 (12.5)	0 (0.0)		0 (0.0)	0 (0.0)	
Others	1 (6.2)	1 (5.3)		1 (7.7)	1 (10.0)	
Multiple	2 (12.5)	1 (5.3)		2 (15.4)	0 (0.0)	
Bupropion	22 (19.8)	112 (16.9)	0.531	19 (19.0)	34 (17.0)	0.664

Values are presented as mean ± standard deviation, median [interquartile range], or number (%).

PHQ-9, Patient Health Questionnaire-9; MMSE, Mini-Mental State Examination; mRS, modified Rankin Scale; NIHSS, National Institutes of Health Stroke Scale; IV, intravenous; tPA, tissue plasminogen activator; IAT, intra-arterial thrombectomy; END, early neurological deterioration; RH, rehabilitation; SSRI, selective serotonin reuptake inhibitor; SNRI, serotonin-norepinephrine reuptake inhibitor.

After 1:2 PSM, a total of 300 patients were included in the matched cohort (100 for PHQ-9 positive and 200 for PHQ-9 negative). The matched cohort was well balanced in terms of standardized differences (Supplementary Table 1), and observed covariates according to PHQ-positive (Table 1).

### Factors associated with patient health questionnaire-9 positivity

Patients with PHQ-9 positivity were younger, had a history of cancer more commonly, a longer length of hospital stay, and a higher NIHSS score at admission (Table 1). The median time interval between END and PHQ-9 screening was 1 day (IQR 0–2.0 days).

In univariable logistic regression analyses (Table 2), a history of cancer or END was associated with PHQ-positivity. In the multivariable logistic regression analysis (Table 2), younger age, history of cancer, and END were independently associated with PHQ-9 positivity.

**Table 2:** Predictors for PHQ-9 positive.

	**Univariable**		**Multivariable**	
	**OR (95% CI)**	***P* value**	**OR (95% CI)**	***P* value**
Demographic/Social variables
Age, years	0.99 (0.97–1.00)	0.078	0.98 (0.97–1.00)	0.024
Sex, female	1.28 (0.85–1.92)	0.232	1.33 (0.88–2.01)	0.176
Education				
Nil	Ref			
Primary	0.74 (0.35–1.54)	0.421		
Secondary and above	0.57 (0.31–1.06)	0.076		
Medicaid	1.75 (0.69–4.44)	0.239		
Right handedness	0.80 (0.35–1.86)	0.606		
MMSE	0.98 (0.93–1.02)	0.317		
Medical History				
Hypertension	1.10 (0.69–1.75)	0.700		
Diabetes	1.49 (0.99–2.26)	0.056		
Dyslipidemia	1.03 (0.69–1.56)	0.875		
Previous stroke	0.85 (0.51–1.43)	0.549		
Ischemic heart disease	0.90 (0.47–1.71)	0.742		
Current smoker	1.13 (0.68–1.88)	0.632		
Atrial fibrillation	0.93 (0.54–1.61)	0.805		
Cancer	2.10 (1.30–3.40)	0.003	2.23 (1.37–3.64)	0.001
Body mass index	1.01 (0.95–1.07)	0.745		
Prestroke mRS > 2	1.74 (0.56–5.37)	0.339		
Stroke variables				
Right hemisphere	1.08 (0.72–1.62)	0.704		
NIHSS at admission	1.03 (0.97–1.10)	0.303		
IV tPA	0.80 (0.37–1.72)	0.561		
IAT	0.37 (0.11–1.22)	0.103		
END	1.70 (1.09–2.65)	0.020	1.69 (1.07–2.65)	0.023

PHQ-9, Patient Health Questionnaire-9; OR, Odds ratio; CI, confidence interval; MMSE, Mini-Mental State Exam; mRS, modified Rankin Scale; NIHSS, National Institutes of Health Stroke Scale; IV, intravenous; tPA, tissue plasminogen activator; IAT, intra-arterial thrombectomy; END, early neurological deterioration.

### Factors associated with poor functional outcome at 3 months

**Figure 2:**
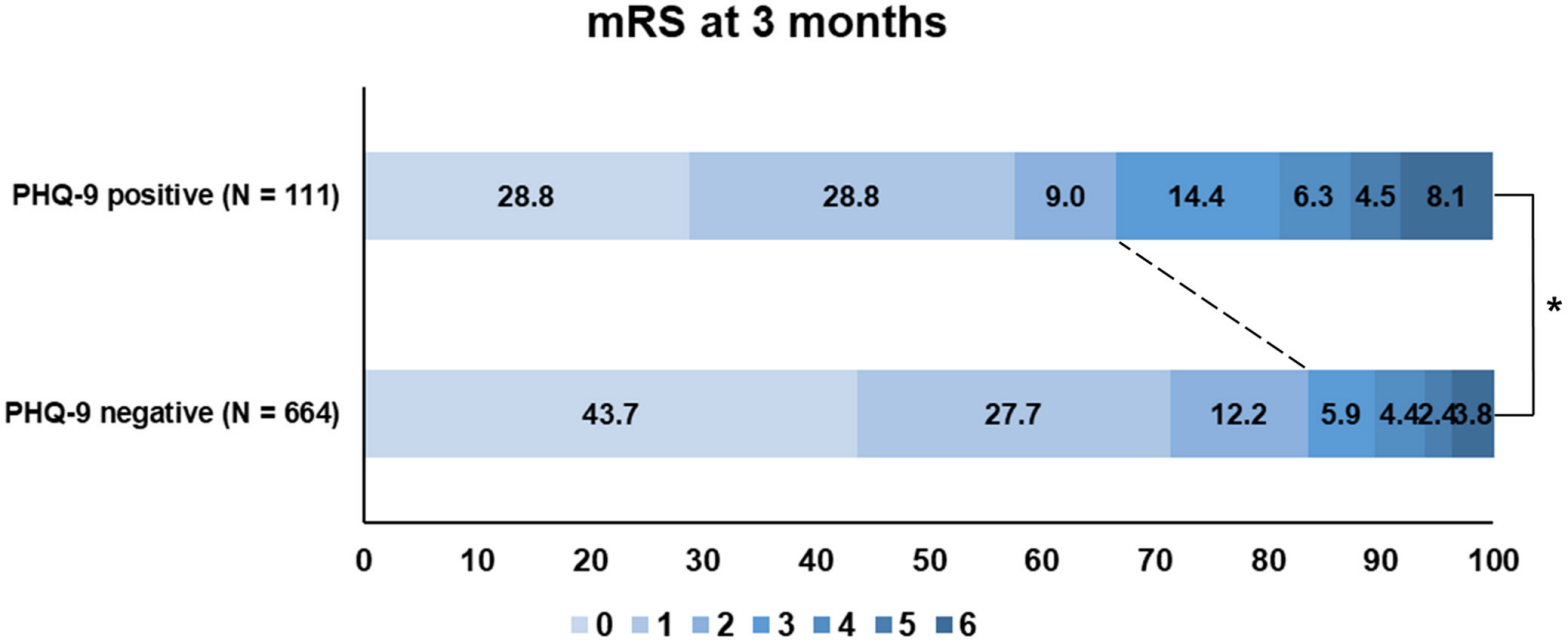
Functional outcome (modified Rankin Scale) at 3 months according to the PHQ-9 status. PHQ-9, Patient Health Questionnaire-9; mRS, modified Rankin Scale.

Patients with PHQ-9 positivity more frequently had poor functional outcomes (mRS score of >2) at 3 months than those who were PHQ-9 negative (33.3 vs. 16.4%; *P* < 0.001; Figure 2). When we additionally trichotomized PHQ-9 results (<5, negative; 5–9, mild; >9, moderate to severe), patients with more severe PHQ-9 results had worse functional outcomes at 3 months (*P* = 0.001; Supplementary Figure 1).

In the univariable logistic regression analysis (Supplementary Table 2), PHQ-9 positivity was associated with poor functional outcomes at 3 months (Odds ratio [OR] 2.55, 95% confidence interval [CI] 1.63–3.97, *P* < 0.001). In the multivariable logistic regression analysis after adjustment for age, sex, and variables that showed *P* < 0.05 in univariable analyses (Table 3), PHQ-9 positivity showed a 2.6-fold higher risk of poor functional outcome at 3 months (OR 2.58; 95% CI 1.26–5.28; *P* = 0.009; Table 3). When the PHQ-9 score was computed as a continuous variable in the same multivariable model, every increase by one point led to a 1.2-fold higher risk of poor functional outcome at 3 months in the multivariable analysis (OR 1.15; 95% CI 1.06–1.26; *P* = 0.002). The PSM analysis also showed PHQ-9 positivity showed 3.6-fold higher risk of poor functional outcome at 3 months (OR 6.72; 95% CI 1.82–24.78, *P* = 0.004; Table 3). When PHQ-9 was used as a continuous variable, every 1-point increase led to 1.3-fold higher risk of poor functional outcome at 3 months.

**Table 3:** PHQ-9 and poor functional outcome at 3 months.

	Multivariable logistic regression^*^		**Propensity score matching^†^**	
	**OR (95% CI)**	***P* value**	**OR (95% CI)**	***P* value**
*Values as continuous variables*
PHQ-9, per 1 increase	1.15 (1.06–1.26)	0.002	1.31 (1.07–1.60)	0.009
*Values as categorical variables*
PHQ-9 positive	2.58 (1.26–5.28)	0.009	6.72 (1.82–24.78)	0.004

* Adjusted for age, sex, and variables (P < 0.05) in univariable analyses (Supplementary Table 2).

† Adjusted for length of hospital stay, NIHSS at discharge, mRS at discharge >2, home discharge, and antidepressant except bupropion.

**Figure 3:**
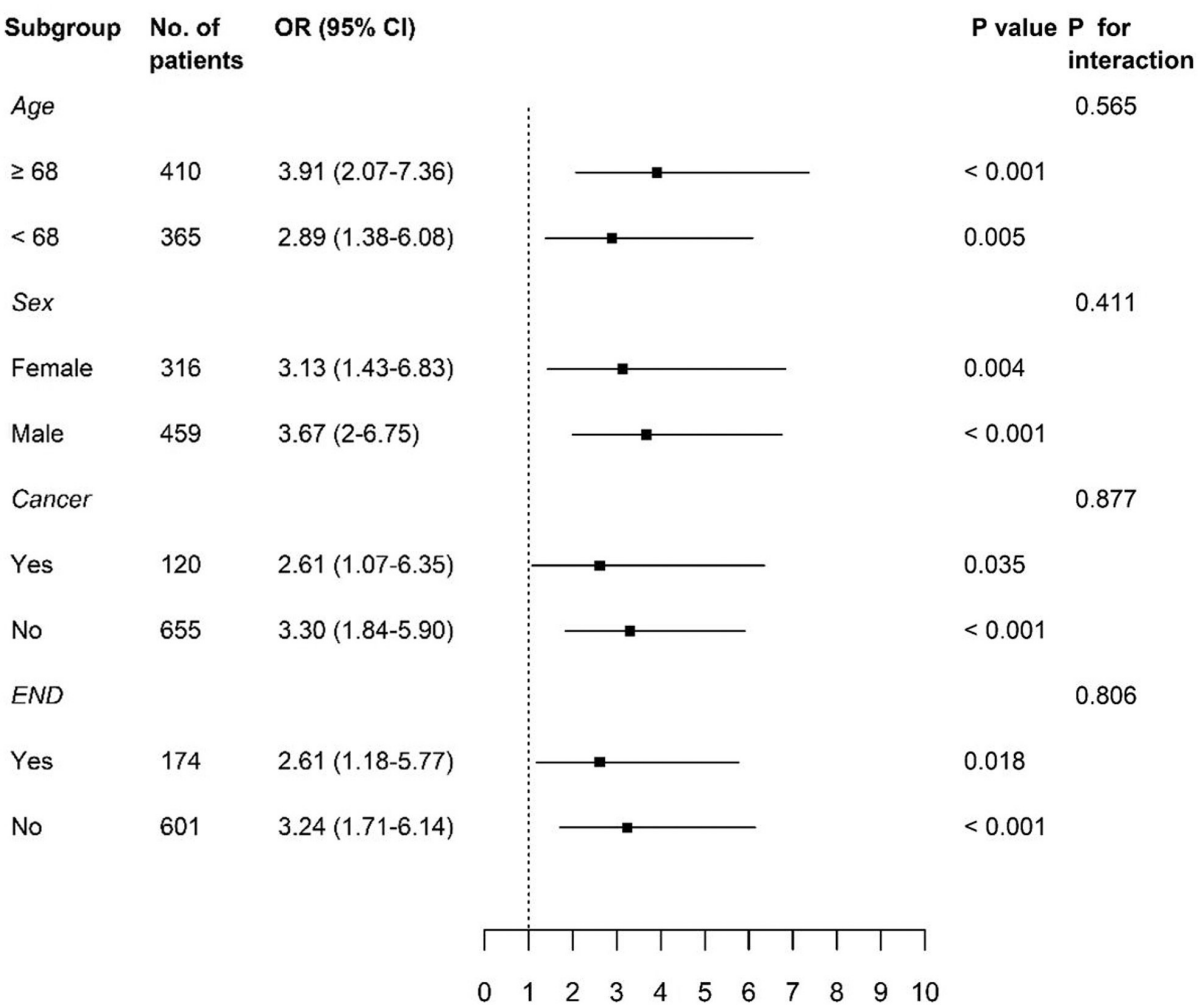
Subgroup analyses of adjusted odds ratios of PHQ-9 positive group for poor functional outcome at 3 months. PHQ-9, Patient Health Questionnaire-9; OR, odds ratio; CI, confidence interval; END, early neurological deterioration.

We divided our study population based on the median age of 68 years, sex, and variables, which were significantly associated with the occurrence of PHQ-9 positivity, the presence of cancer, and END. Significant associations between PHQ-9 positivity and poor functional outcome at 3 months were consistently observed in each subgroup (Figure 3).

## Discussion

In this study, we showed that approximately 14% of patients with acute ischemic stroke or TIA had early PSD based on PHQ-9 assessment. History of cancer and occurrence of END were independent factors for early PSD. Furthermore, early PSD, evaluated using the PHQ-9 during hospitalization for acute ischemic stroke, was independently associated with poor functional outcomes.

PSD was independently associated with short-term functional outcomes at 3 months in this study. This is compatible with previous studies, which showed the association of PSD with worse functional outcomes after stroke (2, 3, 21). Although the pathophysiology of PSD is poorly understood, PSD might limit participation in rehabilitation, directly decrease physical, social, and cognitive function, and perhaps affect the biological process of neuroplasticity (2, 22). Some may argue that early PSD simply reflects a psychological response to a new disability, rather than true PSD (23). A previous study even showed that the timing of assessments for PSD could affect the prevalence of PSD (2). However, in this study, early PSD was independently associated with functional outcome at 3 months, even after adjustments for a diverse range of variables regarding neurologic deficit: NIHSS at admission and at discharge, END, and mRS at discharge. END, which was independently associated with early PSD, was not associated with functional outcome at 3 months when entered into the multivariable model with PHQ-9 positivity. PHQ-9 was consistently associated with poor functional outcomes at 3 months in the subgroup analyses. The importance of early PSD is also highlighted as a requirement for Joint Commission-sponsored Comprehensive Stroke Center certification in the United States (5). Furthermore, a previous study showed that both early PSD (in 2 weeks) and chronic PSD (at 1 year) were associated with worse long-term cardio-cerebrovascular outcomes during 12-year follow-up (21).

In our study, history of cancer and END were independently associated with early PSD. Younger age was not associated with early PSD in univariable analysis, but associated with early PSD in multivariable analysis. Although there are conflicts in the association between age and PSD (2, 3), younger age was suggested to be associated with early PSD (11, 24). Interestingly, our study showed that a history of cancer was associated with PSD. Depression is a common complication in cancer survivors, affecting up to one-fourth of cancer patients (25). Furthermore, depression is even suggested to be associated with increased incidence of cancer and mortality (26). With the rise in the survival rate of patients with cancer, the number of patients experiencing stroke is also escalating and warrants better care for these patients (27). Interestingly, early PSD was associated with END, not NIHSS score at admission. In previous studies, the association between NIHSS score at admission and early PSD was usually investigated; hence, the role of END in early PSD was not known (11, 12, 24). This might suggest that patients with worsened neurologic deficit during admission have a higher chance of having depression and a need for screening of the development or management for PSD. We also investigated a diverse range of factors, including education level, MMSE socioeconomic status (Medicaid), right handedness, and right hemispheric lesion, which have been suggested to be associated with PSD (2, 3). However, they were not associated with early PSD in this study.

This study had several strengths. It was conducted based on registry data, which included extensive and thorough data. Pre-morbid depression status, antidepressant use on admission, and antidepressant use at discharge, including the type of drug, were also evaluated. All admitted patients were systematically screened for PHQ-9 (5). If PHQ-9 could not be performed, the classified reason was recorded in a consecutive manner.

This study has several limitations. First, PHQ-9 positivity indicates not only early PSD, but a depressive tendency as well, which was maintained before stroke, as detected based on the frequency in the preceding 2 weeks of each question in the PHQ-9. Hence, in a previous study, the frequency since hospitalization of each question in the PHQ-9 was evaluated, although it had not been previously validated in the stroke population (11). Second, there may still be the argument regarding the clinical meaning of early PSD defined using the PHQ-9 in the acute period (23). Early screening of depression in hospitalized patients for stroke was investigated in several small studies, especially using the PHQ-9 (11–13), which, being a self-administered questionnaire was easily applicable; PHQ-9 was also used in this study. Future studies based on depression screening or confirmative examination in both acute and chronic periods of stroke might further increase our understanding of early PSD. Third, although it was suggested that there was association between medical adherence and depression, the association between medical adherence and early PSD could not be investigated in this study (28). Fourth, the results of this study should be interpreted carefully, because the study population had relatively mild stroke symptom and were frequently treated with bupropion for anti-smoking purpose.

## Conclusions

Early PSD was associated with worse short-term functional outcomes. Considering the high prevalence of PSD, its early identification could offer opportunities for further questioning and exploration of symptoms, as well as interventions.

## Data availability statement

The raw data supporting the conclusions of this article will be made available by the authors, without undue reservation.

## Ethics statement

The studies involving human participants were reviewed and approved by Institutional Review Board of Severance Hospital, Yonsei University Health System. Written informed consent for participation was not required for this study in accordance with the national legislation and the institutional requirements.

## Author contributions

MB: conception and design, collection, analysis, interpretation of data, and drafting of the manuscript. HyuL, IL, JH, HN, and HyeL: analysis and interpretation of the data and manuscript preparation. YK: conception and design, analysis and interpretation of data, drafting of the manuscript, and revising it critically for important intellectual content. All authors contributed to the article and approved the submitted version.

## Funding

This study was supported by a faculty research grant of Yonsei University College of Medicine (6-2020-0202 and 6-2019-0191) and a grant of the Korea Health Technology R&D Project through the Korea Health Industry Development Institute (KHIDI), funded by the Ministry of Health & Welfare, Republic of Korea (HC19C0028).

## Conflict of interest

The authors declare that the research was conducted in the absence of any commercial or financial relationships that could be construed as a potential conflict of interest.

## Publisher's note

All claims expressed in this article are solely those of the authors and do not necessarily represent those of their affiliated organizations, or those of the publisher, the editors and the reviewers. Any product that may be evaluated in this article, or claim that may be made by its manufacturer, is not guaranteed or endorsed by the publisher.
