# Supplementary material for: Early depression screening and short-term functional outcome in hospitalized patients for acute ischemic stroke
Source: Front Neurol. 2022 Aug 5;13:950045. doi: 10.3389/fneur.2022.950045 (PMC9389070; doi:10.3389/fneur.2022.950045)
Supplement: Supplementary file 1 [file Data_Sheet_1.docx]

Supplementary Material

# Supplementary Figures and Tables

## Supplementary Figures

**
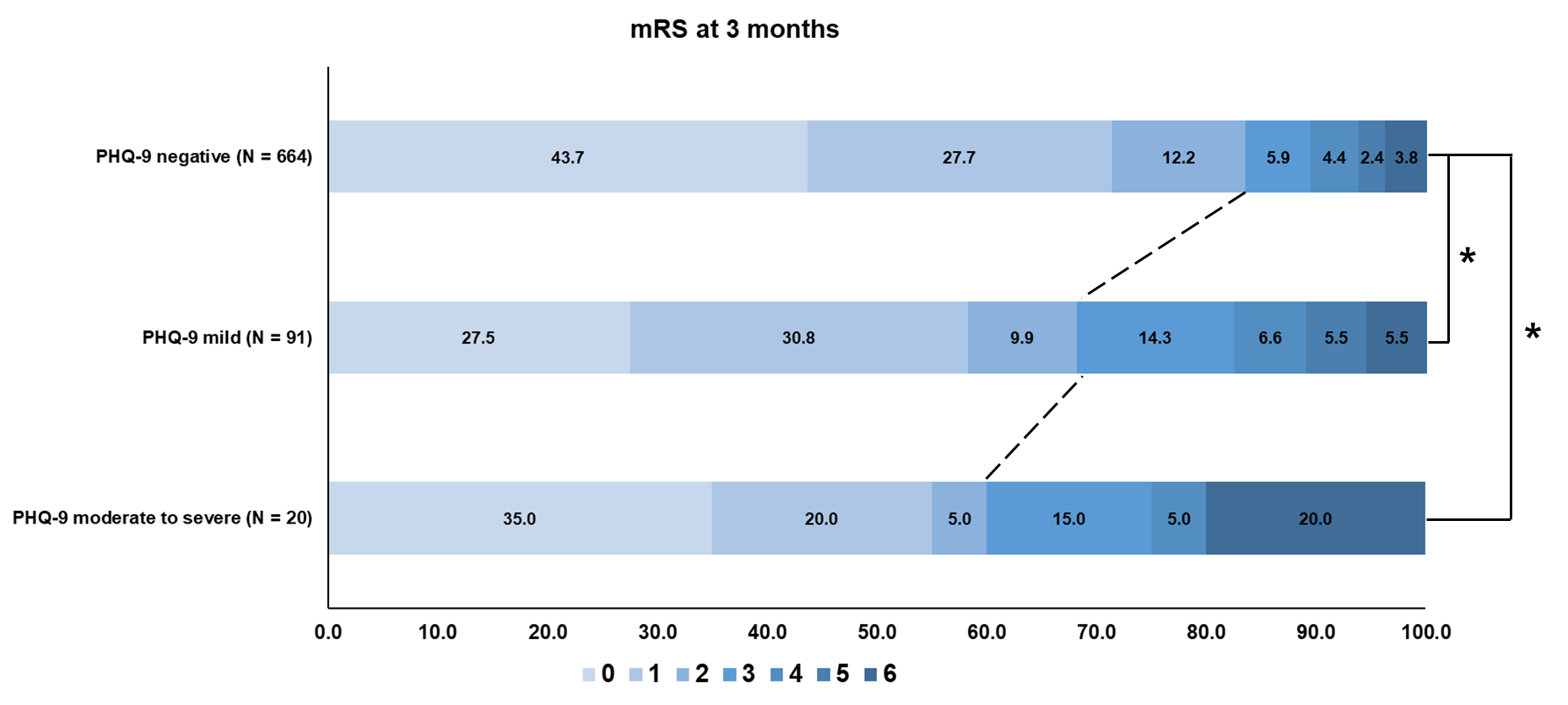
**

**Supplementary Figure 1.** **Functional outcome (modified Rankin Scale) at 3 months according to the trichotomized degree of PHQ-9**

PHQ-9, Patient Health Questionnaire-9.

## Supplementary Tables

**Supplementary Table 1. Standardized differences after propensity score matching**

|  | Standardized difference after matching |
| --- | --- |
| *Demographic/Social variables* |  |
| Age, years | 0.039 |
| Sex, female | 0.040 |
| Education |  |
| Nil | 0.059 |
| Primary | 0.150 |
| Secondary and above | 0.171 |
| Medicaid | 0.021 |
| Right handedness | 0.184 |
| MMSE | 0.057 |
| *Medical History* |  |
| Hypertension | 0.023 |
| Diabetes | 0.023 |
| Dyslipidemia | 0.010 |
| Previous stroke | 0.138 |
| Ischemic heart disease | 0.036 |
| Current smoker | 0.013 |
| Atrial fibrillation | 0.014 |
| Cancer | 0.109 |
| Body mass index | 0.090 |
| Prestroke mRS > 2 | 0.085 |
| Stroke variables |  |
| Right hemisphere | 0.010 |
| NIHSS at admission | 0.040 |
| IV tPA | 0.041 |
| IAT | <0.001 |
| END | 0.045 |

MMSE, Mini-Mental State Exam; mRS, modified Rankin Scale; NIHSS, National Institutes of Health Stroke Scale; IV, intravenous; tPA, tissue plasminogen activator; IAT, intra-arterial thrombectomy; END, early neurological deterioration.

**Supplementary Table 2. Logistic regression for poor functional outcome at 3 months**

|  | Univariable |  | Multivariable |  |
| --- | --- | --- | --- | --- |
|  | OR (95% CI) | P value | OR (95% CI) | P value |
| Demographic/Social variables | | | | |
| Age, years | 1.06 (1.04–1.07) | <0.001 | 1.05 (1.02–1.08) | 0.001 |
| Sex, female | 0.91 (0.63–1.32) | 0.636 | 0.77 (0.41–1.45) | 0.416 |
| Education |  |  |  |  |
| Nil | Ref |  | Ref |  |
| Primary | 0.46 (0.24–0.89) | 0.020 | 0.53 (0.19–1.49) | 0.230 |
| Secondary and above | 0.41 (0.24–0.70) | 0.001 | 1.27 (0.49–3.32) | 0.623 |
| Medicaid | 2.65 (1.19–5.91) | 0.018 | 2.00 (0.57–7.05) | 0.279 |
| Right handedness | 0.96 (0.43–2.11) | 0.910 |  |  |
| MMSE | 0.83 (0.80–0.87) | <0.001 | 0.91 (0.84–0.98) | 0.009 |
| Medical History |  |  |  |  |
| Hypertension | 2.09 (1.30–3.38) | 0.002 | 1.14 (0.56–2.3) | 0.726 |
| Diabetes | 2.33 (1.61–3.36) | <0.001 | 2.49 (1.39–4.47) | 0.002 |
| Dyslipidemia | 1.04 (0.72–1.50) | 0.835 |  |  |
| Previous stroke | 1.91 (1.27–2.87) | 0.002 | 2.08 (1.07–4.05) | 0.031 |
| Ischemic heart disease | 1.54 (0.93–2.57) | 0.097 |  |  |
| Current smoker | 0.54 (0.31–0.92) | 0.025 | 1.44 (0.63–3.27) | 0.387 |
| Atrial fibrillation | 1.56 (1.00–2.44) | 0.048 | 0.95 (0.45–2.02) | 0.897 |
| Cancer | 2.59 (1.68–3.99) | <0.001 | 2.74 (1.38–5.42) | 0.004 |
| Body mass index | 0.94 (0.89–0.99) | 0.026 | 0.93 (0.85–1.01) | 0.070 |
| Pre-stroke mRS >2 | 16.57 (5.37–51.14) | <0.001 | 4.58 (1.04–20.22) | 0.045 |
| Stroke variables |  |  |  |  |
| Right hemisphere | 1.79 (1.23–2.62) | 0.002 | 1.03 (0.59–1.81) | 0.922 |
| NIHSS at admission | 1.30 (1.22–1.38) | <0.001 | 1.03 (0.92–1.15) | 0.573 |
| IV tPA | 1.39 (0.77–2.52) | 0.271 |  |  |
| IAT | 2.46 (1.32–4.56) | 0.004 | 0.68 (0.20–2.25) | 0.523 |
| END | 4.28 (2.91–6.31) | <0.001 | 0.72 (0.34–1.50) | 0.376 |
| Length of hospital stay, days | 1.17 (1.12–1.23) | <0.001 | 1.00 (0.93–1.07) | 0.957 |
| NIHSS at discharge | 1.69 (1.53–1.85) | <0.001 | 1.28 (1.09–1.49) | 0.003 |
| mRS at discharge > 2 | 24.80 (15.78–38.96) | <0.001 | 4.36 (2.07–9.18) | <0.001 |
| Home discharge | 0.05 (0.04–0.09) | <0.001 | 0.21 (0.10–0.43) | <0.001 |
| All antidepressant | 0.90 (0.57–1.41) | 0.640 |  |  |
| Antidepressant except bupropion | 4.47 (2.24–8.91) | <0.001 | 0.62 (0.21–1.81) | 0.383 |
| PHQ-9 positive | 2.55 (1.63–3.97) | <0.001 | 2.58 (1.26–5.28) | 0.009 |

OR, odds ratio; CI, confidence interval; MMSE, Mini-Mental State Exam; mRS, modified Rankin Scale; NIHSS, National Institutes of Health Stroke Scale; IV, intravenous; tPA, tissue plasminogen activator; IAT, intra-arterial thrombectomy; END, early neurological deterioration; PHQ-9, Patient Health Questionnaire-9.
